# Targeting carbon for crop yield and drought resilience

**DOI:** 10.1002/jsfa.8501

**Published:** 2017-08-03

**Authors:** Cara A Griffiths, Matthew J Paul

**Affiliations:** ^1^ Plant Science, Rothamsted Research Harpenden Hertfordshire AL5 2JQ UK

**Keywords:** sucrose, trehalose 6‐phosphate, drought, yield potential, resilience

## Abstract

Current methods of crop improvement are not keeping pace with projected increases in population growth. Breeding, focused around key traits of stem height and disease resistance, delivered the step‐change yield improvements of the green revolution of the 1960s. However, subsequently, yield increases through conventional breeding have been below the projected requirement of 2.4% per year required by 2050. Genetic modification (GM) mainly for herbicide tolerance and insect resistance has been transformational, akin to a second green revolution, although GM has yet to make major inroads into intrinsic yield processes themselves. Drought imposes the major restriction on crop yields globally but, as yet, has not benefited substantially from genetic improvement and still presents a major challenge to agriculture. Much still has to be learnt about the complex process of how drought limits yield and what should be targeted. Mechanisms of drought adaptation from the natural environment cannot be taken into crops without significant modification for the agricultural environment because mechanisms of drought tolerance are often in contrast with mechanisms of high productivity required in agriculture. However, through convergence of fundamental and translational science, it would appear that a mechanism of sucrose allocation in crops can be modified for both productivity and resilience to drought and other stresses. Recent publications show how this mechanism can be targeted by GM, natural variation and a new chemical approach. Here, with an emphasis on drought, we highlight how understanding fundamental science about how crops grow, develop and what limits their growth and yield can be combined with targeted genetic selection and pioneering chemical intervention technology for transformational yield improvements. © 2017 The Authors. *Journal of The Science of Food and Agriculture* published by John Wiley & Sons Ltd on behalf of Society of Chemical Industry.

## INTRODUCTION

### The problem of drought

One of the most important and widespread environmental stresses that affect plant productivity is drought. Drought is for the most part caused by limitations in rainfall, which changes at the hemispheric, continental, regional and even local levels. It is likely that the intensity and the frequency of drought is rising across many regions.^1^, ^2^ Future changes in drought events, particularly in arid and semi‐arid regions, are likely to increase land degradation.^3^ Taking a wider view, drought events can cause regional catastrophes and are considered as a deadly environmental disaster, particularly when drought occurs in a region already afflicted with low water quality, a reliance on rain‐fed crops and high demands for food consumption. A drought event in East Africa caused the deaths of 450 000 people in Sudan and Ethiopia in 1984. More recently, in 2011, extreme drought in Somalia and Ethiopia triggered the movement of 380 000 refugees to neighbouring countries and a requirement of humanitarian aid for ten million people.^4^ Drought events in these regions are reoccurring every decade.^5^ As a result of the frequency of catastrophic drought events and the general water scarcity in these areas, farmers tend to grow drought‐resistant crops, such as sorghum, cassava, millet and cowpea. In 2008, a global food crisis was caused in part by drought and, again in 2012, drought strongly affected crop yields in the USA, which exports 53% of the world's maize and 43% of soya.

Crop species can suffer up to a 50% yield loss if a drought event occurs at the reproductive growth stage^6^, ^7^ and these drought events are often further complicated by heat and other environmental stresses, such as salinity, which often occur in tandem. Drought can also alter plant nutrition and a striking example of this occurs in cassava and sorghum, which are high in cyanide content when drought‐stressed.^8^, ^9^ Keeping the focus on yields in this review, a drought risk assessment was completed for world crop production by integrating historical crop yields and meteorological drought. Strikingly, a prediction of drought‐related yield reduction for major crops will increase by >50% by 2050, and by almost 90% by 2100.^10^ The frequency of drought events is increasing^11^, ^12^ and research efforts should be focussed on making current staple crops more resilient to drought, particularly the main food security crops wheat, maize and rice.

## DROUGHT TOLERANCE STRATEGIES IN THE NATURAL ENVIRONMENT

Plants have evolved over time to resist drought via morphological and physiological changes, often involving osmotic adjustments and the regulation of signalling cascades. When responding to water‐deficit, plants use multiple strategies to preserve optimal relative water content (RWC) in active tissues. Plants utilise mechanisms such as stomatal closure, increasing leaf cuticle thickness and activation of root growth. These traits are considered to be associated with a drought avoidance strategy, where the plant will actively retain leaf water and seek new sources of water deeper in the soil. Furthermore, some plants have evolved mechanisms whereby osmoprotectants, reactive oxygen species (ROS) scavengers and antioxidants are accumulated for cellular protection during water‐deficit. These are considered traits of drought tolerance because the plant will adjust cellular metabolism to maintain homeostasis when RWC is lower.^13^, ^14^, ^15^ Astonishingly, some plants can tolerate complete desiccation during drought stress and will go into a state of suspended animation until re‐watering. These plants are desiccation tolerant, commonly known as resurrection plants.^16^

The isolation and characterisation of the molecular mechanisms involved in these stress tolerance pathways has been a strong focus in plant research over the past 20–30 years. Published work has shown that drought avoidance mechanisms include changes in root architecture, photosynthesis and leaf traits, whereas drought tolerance mechanisms include adjustments in osmolytes, phytohormones, chlorophyll and antioxidants.^17^ Early studies of root growth during drought stress in soybean showed that during water deficit, soybean roots grew deeper into the soil (> 0.6 m depth) than those of well‐watered plants (< 0.6 m depth),^18^ presumably to access water in deep soil. This response was shown to be positively correlated with varying degrees of drought avoidance in a later study on tepary beans.^19^ It is a general trend that root growth is increased during water‐deficit in drought avoidant plants. In some cases, the root length is ten times larger than the shoot.^20^ In general, there is an increase in root:shoot ratio during drought^21^; however, severe drought will reduce root growth capacity. The root not only searches for water in deep soil during drought, but also is integral for root to shoot signalling under these conditions, presumably to decrease leaf growth and water loss. Abscisic acid (ABA), cytokinins, ethylene precursors and malate have been implicated in root/shoot signalling.^22^

### Stomata and leaf traits

Stomata play a key role in drought. In drought avoidant plants, the stomata remain as closed as possible to prevent loss of water. Under optimal growth conditions, the stomata open and close to allow for water and CO_2_ exchange. Because drought disrupts this process, it can reduce the amount of photosynthesis needed for plant growth.^23^ Plants have developed two supplementary photosynthetic pathways, C_4_ and crassulacean acid metabolism (CAM), which confer some benefit under drought. In C_4_ photosynthesis, a metabolic pump is used to concentrate CO_2_ in bundle sheath cells, then carbon fixation is performed in mesophyll cells separately.^24^ The CAM cycle allows for the opening of stomata for CO_2_ absorption and fixation at night, meaning that the stomata remain closed during the day. It is typical of drought‐tolerant plant species to utilise C_4_ and CAM photosynthetic processes because this combination is more water use efficient.^25^ By contrast, in C_3_ photosynthesis, the stomata are open during the day for absorption of CO_2_ and fixation and remain closed at night.^23^ Some species are facultative CAM plants, which switch from C_3_ to CAM during periods of drought.^26^, ^27^

Other mechanisms that plants utilise in drought‐avoidance are associated with leaf traits. Under severe water‐deficits, plants utilise many passive and active mechanisms to avoid excess water loss. For example, leaves will begin to wilt, roll and, in some cases, senesce and drop to preserve water and prioritise metabolism to other younger leaves at a loss of turgor pressure.^28^ In some cases, leaves will also expand, becoming perpendicular to radiation in an attempt to reduce exposure.^29^ Plants that are considered drought resistant tend to have differing xenomorphic structures such as increased trichomes, small and dense stomata, thick cuticle epidermis and smaller, thicker leaves. This is not an exhaustive list; however, each trait is able to reduce water loss and radiation exposure to allow the plant to survive drought stress.

### Phytohormones

The roles of phytohormones in drought stress signalling and plant responses have for the most part centred on ABA; however, recent literature has uncovered roles of hormones that work synergistically with ABA in a variety of stress responses. ABA co‐ordinates drought stress signalling pathways (in addition to cold and salt stress) by activating genes involved in adaptation responses, osmotic adjustment, root hydraulic conductivity, root/shoot growth and ion compartmentalisation.^30^ In addition, ABA reduces the transpiration rate,^31^ which helps reduce water loss. The biosynthesis of ABA during stress is primarily localised to the vascular tissues and guard cells.^32^ ABA then undergoes intercellular transport via two ABC (ATP‐binding cassette) transporters,^33^ as well as nitrate transporters into neighbouring tissues.^34^ In addition to ABA, methyl jasmonate has been shown to accumulate in drought‐stressed tissue of rice, which stimulates the production of ABA leading to grain yield loss.^35^ An increase in intracellular concentration of jasmonic acid has also been shown in pear leaves during water stress, which leads to an increase in the production of the osmoprotectant, betaine.^36^ Strigolactone (SL), a hormone most commonly associated with branching and its relationship with auxin, has also been implicated in drought stress signalling as a positive regulator. It was shown that cross‐talk between SL and ABA mediated stomatal closure to reduce transpiration rates under drought, in addition to showing SL‐induced gene expression pattern changes related to ABA and cytokinin responses.^37^

### Osmotic adjustment and cell protection

Other molecular mechanisms involved in plant responses to water deficit include those that allow for osmotic adjustment, namely the accumulation of osmolytes and stabilising proteins. The protection of cellular components during dehydration is enhanced by the up‐regulation of such compounds such as non‐reducing sugars, amino acids, proteins, alkaloids and polyols. Molecules such as betaine, proline and glycine are able to reduce water efflux from cells and stabilise cytoplasmic constituents.^38^ It has been suggested that the accumulation of these osmolytes creates a preferential exclusion, where the osmolytes are excluded from the protein surface, keeping the protein preferentially hydrated.^39^ Soluble carbohydrates are synthesised during drought stress to aid in cellular protection by stabilising membranes and maintaining turgor. Generally, soluble carbohydrate accumulation will occur in tandem with proline and anthocyanin accumulation, aiding the protection against ROS in the first instance.^40^ The disaccharide trehalose has been implicated in drought tolerance processes in several studies.^41^, ^42^, ^43^ Under drought stress conditions, the accumulation of trehalose potentially aids in the stabilisation of phospholipid membranes, proteins and nucleic acids,^17^ although, in most plants, insufficient trehalose accumulates to enable this function.

The induction of a large set of specific proteins are known to reduce and prevent damage to cells at low water contents.^44^ These protective proteins include chaperones, proton‐regulated ATPases and some proteins with enzymatic functions such as aldehyde dehydrogenase, heat shock proteins and late embryogenesis abundant (LEA) proteins. LEA proteins are a major representative of protective proteins that are generally hydrophilic, and also have an amino acid composition mostly devoid of cysteine and tryptophan. In many cases, the function of LEA proteins is not known.^45^ LEA proteins are expressed during drought stress and are found in most cell types, accumulating for the most part in plastids and the cytoplasm.^46^ The distribution of LEA protein correlates with a protective function where the LEA proteins may form anchors in a structural network, which stabilises cytoplasmic components during dehydration. It has been suggested that LEA proteins exert their protective role by replacing water to maintain hydration of proteins and other cellular components. LEA proteins may act in conjunction with carbohydrates to perform this role and may also bind to ions, thereby decreasing ion concentration in dehydrated cells. Biochemical evidence is lacking for these hypotheses.^47^

Aerobic metabolism produces ROS by‐products, namely ^1^O_2_, H_2_O_2_, O_2_^–^ and OH^–^. Under normal plant growth conditions, ROS is detoxified using the plant antioxidant defense system. However, during drought, the balance between aerobic metabolism and ROS detoxification is disrupted.^48^ ROS are produced in chloroplasts, peroxisomes, plasma membrane, cell walls and endoplasmic reticulum. The accumulation of ROS in the cell causes lipid peroxidation, DNA damage and protein carbonylation, triggering a cascade of events ultimately leading to cell death.^49^ Both enzymatic and non‐enzymatic responses are utilised by plants to detoxify ROS. This includes (but is not limited to) the enzymes superoxide dismutase, ascorbate peroxidase and catalase, as well as the reducing compounds ascorbic acid, carotenoids and glutathione.^17^ It could be considered that the antioxidant defence system is the plant's first response to drought stress and a finely‐tuned system could ultimately combat the detrimental drought‐induced damage.

## PREVIOUS APROACHES TO IMPROVING CROP DROUGHT TOLERANCE

Drought resistance is a complex trait and no single mechanism can be used in transgenic or breeding approaches to confer drought tolerance throughout the life cycle of the plant. Additionally, the requirements of the agricultural environment are productivity and not just survival. Yield penalties in drought tolerant crops are not acceptable. The ideal solution for agriculture is to combine yield and resistance traits together. In the past 20 years, many approaches to improving drought tolerance in crops have been attempted using transgenic and breeding/selection approaches,^50^, ^51^ with some success in the laboratory, although this is harder to demonstrate in the field, particularly for transgenic approaches, and large breakthroughs have yet to be achieved in the field.

### Breeding for drought

The International Maize and Wheat Improvement Center (CIMMYT) has provided wheat varieties adapted to marginal environments, which have been adopted globally through multi‐environmental testing and collaboration with international breeding programmes.^52^ However, the rate of yield increase is still too low to catch up with the projected 70% increase in demand for wheat by 2050. Much of the yield increase under drought is likely to result from spillover benefits of selection for yield improvement under good growing conditions (e.g. reduced plant height).^53^ The flowering period is a growth stage particularly sensitive to drought. Delayed silking is a side effect of drought and is commonly used as selection in breeding approaches to drought tolerance for maize.^54^ In wheat, a reduced number of days to anthesis and maturity enables the crop to evade terminal drought.^55^ Root angle is a common trait for selecting drought‐tolerant phenotypes where the root angle direct influences root distribution in the soil, allowing for deeper roots to develop and find water.^56^ In soybean, improved nitrogen fixation has been associated with higher yields under drought,^57^ whereas, in Burmuda grass, rhizome production was shown to be closely linked with drought tolerance.^58^, ^59^ Wheat traits of reduced evaporative losses and maintenance of assimilate production seen in leaf rolling and flag leaf persistence are used as selection parameters. High stomatal conductance and transpiration seen as low canopy temperature associated with better water uptake are positively correlated with yield under drought and can also be selected for superior performance.^60^

### Transgenic approaches to drought

There are many examples of transgenic plants that appear to perform better under limiting water availability, although often these are slower growing (and lower yielding) and conserve water because of this.^61^ There are few examples where transgenic approaches have been beneficial for crop performance under limiting water availability in the field environment. In rice, the expression of OsbZIP23, a close homologue to the *Arabidopsis* ABF/AREB, a major regulator in the ABA signalling pathway, was shown to increase drought and salinity tolerance.^62^ In addition, rice over‐expressing OsZIP16 also showed improved drought tolerance.^63^ Further drought tolerance has been shown in plants over‐expressing DREB1A (drought responsive element B1A), a protein belonging to the AP2/ERF transcription factor family. Plants that over‐express this gene have a greater tolerance not only to drought stress, but also salinity and cold stress.^64^ It has been shown that these transgenic lines have increased expression of stress‐related genes,^65^ and also have increased yield compared to the wild‐type under drought conditions.^66^ Commonly among transgenic plants ectopically expressing stress‐related transcription factors, drought tolerance has been also associated with NAC transcription factors,^67^, ^68^ OsDT1/HDG11^69^ and OsMYB2.^70^ The ARGOS gene family functions as part of the negative feedback for ethylene response in plants,^71^, ^72^ and has been shown to enhance drought tolerance in maize; however, yield reductions were seen in cool, humid environments.^73^ In sugar cane, overexpression of a choline dehydrogenase (*beta*) from *Rhizobium meliloti* improved drought tolerance and yield.^74^ Commercially, the only drought‐tolerant crop available is maize, which expresses a cold shock protein (CspB) under a Rice actin promoter that acts to stabilise RNA and aids in the production of growth‐related genes during drought.^75^

An interesting approach to drought tolerance is by modulating carbon allocation and source/sink strength.^76^, ^77^ In wheat, phenotypic analysis has shown that remobilisation and storage of soluble carbohydrates is a promising breeding trait for yield stability.^78^ Most recently, in maize, it was shown that a decrease in the sugar signalling molecule trehalose‐6‐phosphate (T6P) in developing kernels increased yield in both drought‐stressed and well‐watered conditions^79^ showing the power of adjusting source/sink strengths to attain better yielding crops.

## THE TREHALOSE PATHWAY: A SHORT HISTORY OF LEARNING WHAT IT DOES

Interestingly, trehalose was first targeted in biotechnology with a view to increasing its concentration as an osmolyte for drought protection. This did not result in drought tolerant crops through accumulating trehalose but did initiate a period of enlightenment about the role of T6P instead, now known as a powerful sugar signalling molecule, the modification of which allows significant yield benefits in crops. The current proliferation of mechanistic understanding, physiological roles and applications of trehalose metabolism in plants and crops began with studies reported in 1997 and 1998 concerning experiments that heterologously expressed genes for the pathway. First, *Escherichia coli* genes were expressed in plants with interesting effects on growth and development.^80^ Second, newly found plant genes functionally complemented yeast mutants.^81^, ^82^ Unexpectedly, not only did it appear that plants contained a whole new sugar pathway (and another nonreducing disaccharide such as sucrose) but, at the same time, through the effects in transgenic plants, it appeared that it could be a strong regulator of growth and development. Prior to this time, transgenic plants with altered carbon metabolism were characterised by either minimal phenotypes or strongly negative phenotypes.^83^ However, the plants reported in Goddijn *et al*.^76^ and subsequently characterised in greater depth.^84^, ^85^ had robust phenotypes displaying previously unreported traits in metabolically engineered plants of higher growth rates and higher rates of photosynthesis than wild‐type. It appeared that an important mechanism of the regulation of growth and development could be controlled somehow by the trehalose pathway and, given the positive effects, could be utilised in crop improvement. Up until this time, trehalose had been previously detected only in leaves of the desiccation‐tolerant *Myrothamnus flabellifolius*.^86^ Subsequently, using far more sensitive detection methods, trehalose has been detected widely in diverse tissues and plants, mostly in trace amounts reflecting a ubiquitous low flux plant pathway. Research over the following 20 years has fulfilled the promise and vision of the early reports with a number of potentially recent significant developments targeting the pathway for crop improvement. Importantly, this has involved fundamental science in parallel with translation in crops.

Studies on heterologous expression in 1997 and 1998 had shown the potential of the pathway to control growth and development and hence improve crops. How essential was the pathway? Eastmond *et al*.^87^ showed for the first time that trehalose 6‐phosphate synthase 1 (TPS1) was essential for *Arabidopsis* embryo maturation. *tps1* mutants could be subsequently rescued through expressing the *E. coli otsA* encoding TPS.^80^ T6P levels could also be elevated in wild‐type expressing *otsA*.^84^ Significantly the role of T6P rather than trehalose underpinning the indispensability of the pathway was proposed because expression of a trehalose phosphate hydrolase (TPH) and a trehalose phosphate phosphatase (TPP), which both catabolised T6P to different end products (glucose in the case of TPH and trehalose in the case of TPP), produced the same phenotype, which appeared to be trehalose independent.^84^ This study was the first to report specifically the role of T6P being indispensable for carbohydrate utilisation in plants.

A number of laboratoriess have developed technology to measure micromolar amounts of T6P in plants.^88^, ^89^, ^90^, ^91^ Lunn *et al*.^88^ was the first to show a likely causal link between sucrose and T6P, with T6P a signal of sucrose status. In this work, strong responses of T6P to sucrose were found in carbon‐starved *Arabidopsis* seedlings. Similar observations have been made in other tissues such as wheat grain but where both sucrose and T6P levels are far higher. Indeed, in wheat grain endosperm of 119 nmol T6P g^–1^ fresh weight (FW) have been reported^92^ compared to 18 pmol T6P g^–1^ FW in carbon‐starved *Arabidopsis* seedlings.^88^ In *Arabidopsis* seedlings grown under more widely relevant physiologically conditions, T6P levels can reach 10 nmol g^–1^ FW and the sucrose:T6P relationship still holds strongly.^93^ Martinez *et al*.^92^ were the first to document in detail T6P levels in a major crop as a potential mechanism underpinning grain development and hence yield. This not only confirmed the sucrose signal hypothesis, but also showed strong tissue and developmental dependency in the regulation of T6P levels. Tissue and developmental specificity has been an important factor in targeting T6P in crop improvement (see below).

T6P functions in plants and crops as a signal of the availability of sucrose, likely specifically sucrose. T6P responds to sucrose in a highly tissue and developmental manner regulating processes involved in using sucrose to synthesise end‐products involved in growth, development and crop yield. There is strong evidence for the coordination of metabolism with growth and development by T6P^94^, ^95^, ^96^ at the level of gene expression^94^ and post‐translational modification^96^ such that T6P positively regulates processes that use carbon in organic acid and amino acid metabolism,^97^ cell wall synthesis^94^ and starch synthesis,^98^, ^99^ including breakdown of starch and likely protein breakdown under limiting carbon supply and low T6P levels.^94^, ^95^ Interestingly, the translation of feedforward effects into increased growth are strongly dependent on the environment. Nunes *et al*.^93^ showed that expression of genes known to be regulated by T6P increased very similarly in *Arabidopsis* seedlings where sugars increase in tissues as a result of sucrose feeding and concomitant high growth, and at low temperature and low growth. This latter study highlighted the role of T6P in priming gene expression for growth in response to high sugar. Nunes *et al*.^93^ subsequently showed that this priming mediated by T6P enabled growth recovery or the growth spurt after a period of low temperature (i.e. growth was poised through sugar‐induced T6P‐regulated gene expression in anticipation of a return of warmth).

**Figure 1:**
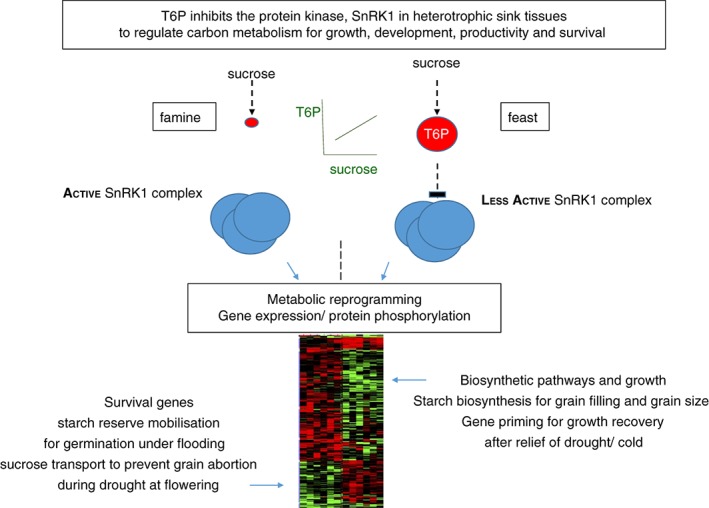
The role of trehalose 6‐phosphate/SnRK1 in plants and crops.

Such widespread effects on metabolism would likely require interaction of T6P with a regulator such as a protein kinase that could regulate post‐translational modification such as protein phosphorylation and gene expression. The findings of Zhang *et al*.^94^ and subsequent support from many other studies,^79^, ^90^, ^100^, ^101^ showing that T6P inhibits SnRK1 in sink and growing tissues to elicit changes in growth, metabolism and development through large changes in expression of genes known to be regulated by SnRK1,^102^ provides a framework for understanding the dramatic effects of T6P in plants and crops (Fig. 1). Indeed, such large‐scale effects mediated through T6P/SnRK1 affect whole plant assimilate distribution and harvest index, which can be targeted to increase crop yields.^79^ However, this mechanism is different in sink and source tissues,^94^, ^97^, ^103^ potentially because of differences in the make‐up of SnRK1 complex in source and sink tissues, developmental regulation of trehalose pathway gene expression with a far stronger expression in sink tissues, and likely additional targets of T6P. Interestingly, in this latter regard, T6P regulates a glucose‐permeable solute channel in the chloroplast outer envelope membrane.^104^ Effects on flowering could also involve other receptors^105^; however, SnRK1 has been shown to affect flowering.^102^, ^106^

There is considerable evidence therefore that T6P regulates metabolic homeostasis in the light of sucrose supply and that this also regulates sucrose homeostasis itself.^107^, ^108^ Anything that can alter the link between sucrose, growth and development is a potential way to improve productivity. Because productivity *per se* (i.e. large numbers of large seeds) does not have high selection pressure in the natural environment, there is understandably room for selection that has not yet reached a limit in crops. The emergence of this major regulatory hub provides the means to achieve this as long as we know how to select and modify the T6P/ SnRK1 mechanism appropriately. Constitutive expression of trehalose pathway genes is unlikely to be successful because of the high cell and developmental nature of regulation, the subtleties of which are lost through constitutive expression. Already, there are striking examples of crop improvement in the three major food security crops (cereals wheat, maize and rice) using three different approaches to modify T6P levels.

## MOVING CARBON FOR YIELD IN DIFFERENT ENVIRONMENTS AND HOW TO TARGET WITH 3 STRATEGIES

Nuccio *et al*.^79^ reported on extensive field trialling of transgenic maize expressing a rice TPP gene driven by a MADS6 promoter. The transgenic progeny were higher, yielding at a whole range of water availabilities during the flowering period, from severe drought through to full irrigation. Drought during the flowering period is a major cause of yield loss in cereals.^109^ It had been proposed that restricted sucrose supply to developing female reproductive structures during drought was a reason for seed abortion.^110^ Given the role of T6P in regulating sucrose utilisation,^84^ targeting T6P levels in reproductive tissue during the flowering period was proposed as a possible means of alleviating seed abortion. Interestingly, T6P levels were reduced by two‐ to three‐fold in female spikelets. High T6P stimulates biosynthetic processes, whereas low T6P stimulates breakdown and survival processes. Nuccio *et al*.^79^ reported higher yield as a result of higher seed numbers that contained more sucrose in developing kernels. Indeed, there was altered assimilate distribution at the whole plant level away from stems toward the ears resulting in improved harvest index. Targeted changes in T6P could result in sucrose movement to where starvation is perceived as one of a number of starvation responses. This is one of very few reports where modification of an intrinsic plant process has been improved through genetic modification. The reason for its success is likely that T6P regulates many genes that coordinate metabolism with growth and development^94^ as a central regulator of source‐sink interactions not yet optimised for yield in crops. Careful targeting of the transgene with a developmental promoter is a second reason for success; many other promoters linked to TPP did not improve yield.^79^ Improving sucrose allocation to developing seeds likely has general utility in improving yield in a range of environments.

A TPP gene was also found to underpin another important trait in rice.^111^ A quantitative trait locus had been identified for superior rice germination under anaerobic conditions. Much wetland rice needs to transplanted because germination is not possible under flooding as a result of anoxia. Transplantation is labour intensive and results in lower yield because of the disruption caused to plant growth. Therefore, direct seeding of rice has been a sought‐after goal. The rice TPP7 gene is expressed in germinating tissues and likely results in localised lower T6P, which acts as a starvation signal resulting in better mobilisation of starch reserves promoting superior germination under anaerobic conditions.

Targeted changes in expression of a TPP gene in active metabolic sink tissues promotes increased seed number in maize, particularly when exposed to drought at flowering^79^ and enhanced germination under flooding.^111^ These effects were achieved by localised decreases in T6P through genetics, which activates starvation responses (sucrose movement, starch mobilisation). However, in different cells, it may be advantageous to increase T6P levels. Indeed, this may have been part of the domestication process because crops wheat and maize contain far higher levels of T6P than wild species such as *Arabidopsis*. The benefits of increasing T6P may come as a result of promoting large seed size and accompanying biosynthetic processes, such as starch synthesis. Up until now, documented genetic increases in T6P have produced small changes in the region of two‐ to three‐fold, using constitutive and inducible promoters.^84^, ^97^ There are no known improved crop traits that have been produced in this way through genetic modification or through natural variants, although it is quite likely that as yet undiscovered natural variation in increased T6P could underpin crop traits. However, because of the strong mechanism of sucrose homeostasis regulated by T6P, it may be difficult to push the limits of T6P accumulation by genetic modification where endogenous regulation is likely to dampen changes in T6P in the long term. Small decreases in T6P may be beneficial^79^, ^111^; yet, to reap benefits of increasing T6P, it may be necessary to push the boundaries further and this may not be possible with GM. GM also requires the testing of numerous promoters that require optimising in different crop systems to be tested, which is a large task. As an alternative, Griffiths *et al*.^99^ pioneered a chemical method to release large short‐term bursts of T6P into plant tissues using signalling precursors. Given the precedent for T6P in activating or priming biosynthetic metabolism,^88^, ^94^, ^97^, ^98^, ^112^ this could be a way of significantly upregulating starch synthesis in seeds, such as wheat grain. Applying T6P precursors during the early grain filling period resulted in more starch in harvested wheat grain and a dramatic increase in seed yield and size of up to 20% in some cases.^99^ A simple explanation is that a large short‐term increase in T6P is sufficient to increase the expression of genes for starch biosynthesis increasing overall sink capacity. This shows that significant gains can be made by increasing sink capacity and that the plant has sufficient photo assimilate available to fill this extra sink capacity through photosynthesis or through mobilisation of other carbohydrate reserves to fill the developing wheat grain. Accompanying the enhanced grain size trait was the finding that droughted wheat plants recovered better upon rewatering after applying T6P precursors 1 day before rehydration. This can be explained because T6P provides generic priming of gene expression for carbon flow into end‐products and this differs depending on the tissue involved. For vegetative wheat plants, priming gene expression for cell wall synthesis required for regrowth could explain the resurrection effect.

Overall, with respect to crop improvement, it may have been that T6P levels have diverged in different cells and tissues to combine adaption to diverse environmental conditions. Activation of famine responses as a result of low T6P would deal with stress in some cells involved in the mobilisation of storage reserves and the movement of sucrose, whereas elevated T6P in sink organs such as seeds will drive a large seed size and the accumulation of end products such as starch. Given the large increases in crop performance in wheat, rice and maize through genetic and chemical intervention approaches, it is likely that there will be further opportunities to increase crop yields through the careful targeting of this pathway in these cereals, as well as in other crops.

## CONCLUSIONS

Water availability remains the most widespread limitation to crop yields and an urgent target in crop improvement programmes. Breeding has produced some advances with new varieties, although there is only one commercially available drought tolerant GM variety. It has not been a straightforward task to improve such a complex trait with single genes. Drought ultimately limits cereal yields by impinging on grain numbers and size. In the future, given that improvements have already been made in crop phenology, height and for better water uptake through selection of stomatal conductance, signalling pathways provide promise for enhancing drought tolerance through their involvement in determining grain number and size. In this review, we have focused on a sugar‐signalling pathway that affects both grain numbers and size. Currently, it would appear that the allocation and utilisation of sucrose and carbon is not optimised in crops to maximise yield and to prevent yield loss under drought. T6P is part of a whole plant sucrose homeostatic mechanism that can alter sucrose allocation and its use in grain for starch synthesis. Remarkably, the same pathway can also improve rice germination under flooding by improving the mobilisation of starch. T6P could therefore feature in crop improvement quite broadly through modification of the carbon metabolism even beyond any obvious applications in yield potential and drought resilience.
